# Healthcare-associated urinary tract infection: epidemiology, burden of disease, and related factors at a teaching hospital in Ho Chi Minh City, Vietnam 2017–2022

**DOI:** 10.1017/ash.2025.102

**Published:** 2025-09-03

**Authors:** Huynh Minh Tuan, Pham Thi Truong Ngan, Nguyen Vu Hoang Yen, Trinh Thi Thoa, Truong Thi Le Huyen, Huynh Hoang Hai, Le Thanh Truyen, Nguyen Thi Minh Khai, Le Thi Yen Nhi, Pham Thi Lan

**Affiliations:** 1 University Medical Center, HCM city; 2 University of Medicine and Pharmacy at HCM city

**Keywords:** Urinary tract infection, Intensive care unit, Healthcare-associated infection

## Abstract

**Introduction:** Urinary tract infection (UTI) is a common healthcare-associated problem. UTI has a lower mortality prevalence than other infections, but it is at high risk of leading to sepsis and increased treatment costs. Therefore, the objective of the study is to describe the epidemiology and burden of disease and determine factors associated with healthcare-associated UTI in the intensive care units (ICUs). **Methods:** A cross-sectional study was conducted on 4.028 patients admitted to the ICU, Neuro Surgical ICU, and ICU - Cardiovascular Surgery Department at a teaching hospital in Ho Chi Minh City from 2017 to 2022. The study collected secondary data through electronic medical records, including age, gender, diagnosis, department, urinary catheter use, urinary catheter retention time, treatment, and urine test results. **Results:** The prevalence of UTI in ICUs was 4.0%, of which CAUTI accounts for the highest prevalence, with the typical pathogen being *E. coli*. The Neuro Surgical ICU had the highest incidence and catheter-used prevalence in ICUs. UTIs were concentrated in people over 80 years old, females, and brain diseases. The length of the hospital stay was long, and the cost of the hospital stay was unaffordable, up to hundreds of millions of VND. The study found factors associated with the prevalence of UTI, such as age, gender, department, diseases, and urinary catheters. Patients with urinary catheters have a 10.98 times higher prevalence of UTI (p < 0.001; PR = 10.98, 95% CI 4.87–24.76) compared to patients without urinary catheters. **Conclusions:** The results of the study demonstrated that UTI remains a burden on the healthcare system, especially in ICUs. Implementing a UTI prevention package for patients with catheters is important. Besides, it is necessary to maintain continuous training for healthcare workers to properly and timely insert, remove, and replace catheters.

